# Efficient Induction of Extrinsic Cell Death by Dandelion Root Extract in Human Chronic Myelomonocytic Leukemia (CMML) Cells

**DOI:** 10.1371/journal.pone.0030604

**Published:** 2012-02-17

**Authors:** Pamela Ovadje, Caroline Hamm, Siyaram Pandey

**Affiliations:** 1 Department of Chemistry and Biochemistry, University of Windsor, Windsor, Ontario, Canada; 2 Windsor Regional Cancer Centre, Windsor, Ontario, Canada; University of Alabama at Birmingham, United States of America

## Abstract

**Background:**

Chronic Myelomonocytic Leukemia (CMML) is a heterogeneous disease that is not only hard to diagnose and classify, but is also highly resistant to treatment. Available forms of therapy for this disease have not shown significant effects and patients rapidly develop resistance early on in therapy. These factors lead to the very poor prognosis observed with CMML patients, with median survival duration between 12 and 24 months after diagnosis. This study is therefore centered around evaluating the selective efficacy of a natural extract from dandelion roots, in inducing programmed cell death in aggressive and resistant CMML cell lines.

**Methodology/Principal Findings:**

To confirm the induction of programmed cell death in three human CMML cell lines, nuclear condensation and externalization of the phosphatidylserine, two main characteristics of apoptosis, were detected using Hoechst staining and annexin-V binding assay. The induction of another mode of cell death, autophagy, was determined using a monodansylcadaverine (MDC) stain, to detect the formation of autophagy vacuoles. The results from this study indicate that Dandelion Root Extract (DRE) is able to efficiently and selectively induce apoptosis and autophagy in these cell lines in a dose and time dependent manner, with no significant toxicity on non-cancerous peripheral blood mononuclear cells. More importantly, we observed early activation of initiator caspase-8, which led to mitochondrial destabilization and the induction of autophagy, suggesting that DRE acts through the extrinsic pathway of apoptosis. The inability of DRE to induce apoptosis in dominant-negative FADD cells, confirms the mechanism of action of DRE in *in vitro* models of CMML.

**Conclusion:**

The results from this study indicate that natural products, in particular Dandelion Root Extract, have great potential, as non-toxic and effective alternatives to conventional modes of chemotherapy available today.

## Introduction

The risk of developing cancer increases with age; nonetheless it can affect all ages [1]. Chronic Myelomonocytic Leukemia (CMML) is a highly aggressive and resistant form of leukemia with a highly variable natural course of progression. The difficulty in diagnosing this disease contributes to its aggressive and resistant nature. In recent years, the World Health Organization (WHO) has classified this disease as both a myeloproliferative disease (MPD) and a myelodysplastic syndrome (MDS) [2], [3]. This classification system has improved diagnosis of CMML, although it can still prove difficult. Even with advances in chemotherapy research, the prognosis for CMML still remains very poor with median survival of 12 to 24 months [3]. The available forms of treatment come with very severe side effects, with patients developing resistance early on during treatment. The only effective form of treatment that has shown significant success in CMML patients is Hematopoietic Stem Cell Transplantation, although very few studies have been done with CMML MDS/MPD [4]. It is therefore of utmost importance to develop more modes of treatment that are not only safe, but also effective in targeting CMML cells specifically.

Natural products have been used for centuries as a source of nutrition, nourishment and for various forms of therapy for a wide range of diseases. Recent reports indicate that over the past sixty years, more than 60% of the approved anti-cancer drugs have been either natural or derived from natural products [5]. *Taraxacum Officinale*, commonly known as Dandelion, are perennial weeds of the Asteracaea family, thought to have originated in central Asia but now known to grow almost anywhere in the world today. Traditional medicine, especially Traditional Chinese Medicine, has encouraged the use of dandelion extracts for the treatment of various diseases, ranging from diarrhoea and digestive diseases to more serious ailments like hepatitis and anorexia [6], [7]. There have been very few scientific studies to ascertain many claims of the use of dandelion extracts in the treatment of diseases, but recently, studies indicate this plant has anti-inflammatory, antioxidant and anti-carcinogenic activities [6]. Our group is studying the anticancer effects of dandelion root extract (DRE), by evaluating its ability to induce physiological programs of cell death in aggressive, resistant CMML cells. Previous work done by our group showed that DRE effectively induced apoptosis in human T cell leukemia (Jurkat) and melanoma cells, by rapidly activating the death-receptor mediated extrinsic pathway of apoptosis [8], [9]. In this study, the efficacy of DRE in more aggressive leukemia cell lines was assessed to determine its selectivity and efficacy in inducing apoptosis/autophagy in CMML cells. Results indicate that DRE effectively induces apoptosis and autophagy in a dose and time dependent manner. The rapid activation of caspase-8, through the activation of the extrinsic pathway of apoptosis, was observed in these cells, comparable to levels found in Jurkat cells. Non-cancerous peripheral blood mononuclear cells (ncPBMCs), treated with dandelion root extract in parallel, were not susceptible to apoptosis, demonstrating the selectivity of dandelion root extract in cell culture. Results from this study indicate that dandelion root extract could potentially represent a novel non-toxic alternative to conventional cancer therapy available today.

## Results

### DRE effectively induces apoptosis in Chronic Myelomonocytic Leukemia cells

**Figure 1:**
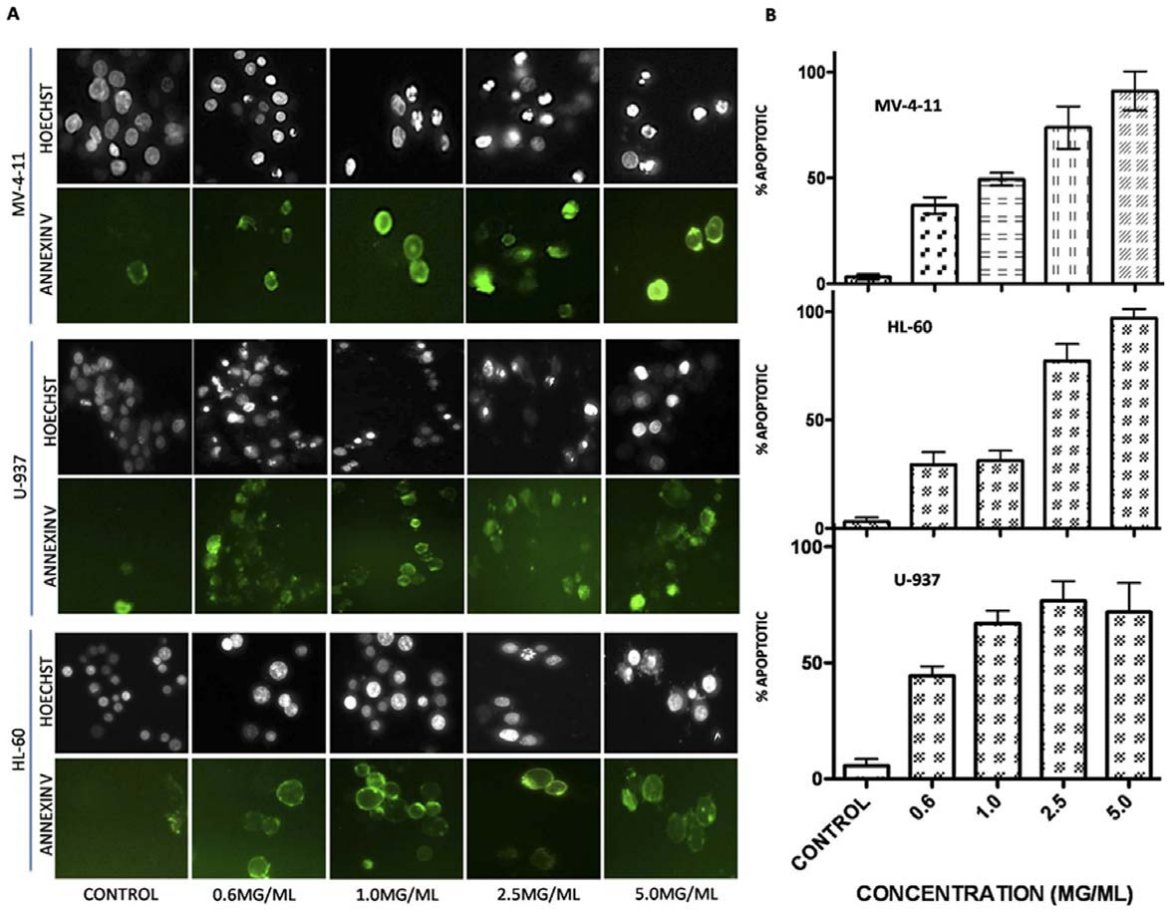
DRE induces apoptosis in CMML cells in a dose dependent manner. MV-4-11, HL-60 and U-937 cells were treated with increasing doses of DRE and analyzed for the induction of apoptosis. (a) Hoechst and Annexin-V staining of DRE-treated CMML cells 48 hours after treatment. Magnification 400×. (b) Manual quantification of three individual experiments of DRE-treated cells, 48 hours after. 6 pictures were obtained for every sample and live versus dead cells were counted to ascertain the percent apoptotic cell count for every sample. Experiment was performed three times and the mean and standard deviation of the three experiments were obtained.

**Figure 2:**
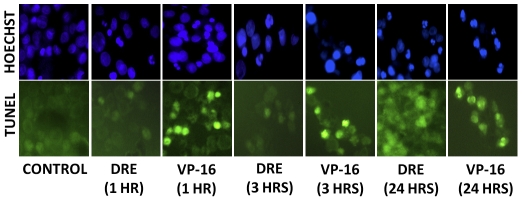
DRE induces late stage DNA damage in MV-4-11 cells. MV-4-11 cells were treated with 1.0 mg/ml DRE and 10 µM VP-16 at the indicated time points to determine if DNA damage was the cause or effect of apoptosis observed in CMML cells. Following treatment, cells were fixed and immunostained with anti-BrdU antibody to observe DNA damage. Increase in TUNEL positive staining corresponds with an increase in levels of DNA damage following treatment. More positively stained cells are observed in the final stages of apoptosis caused by DRE, compared to the DNA targeting drug, VP-16. magnification: 200×.

**Figure 3:**
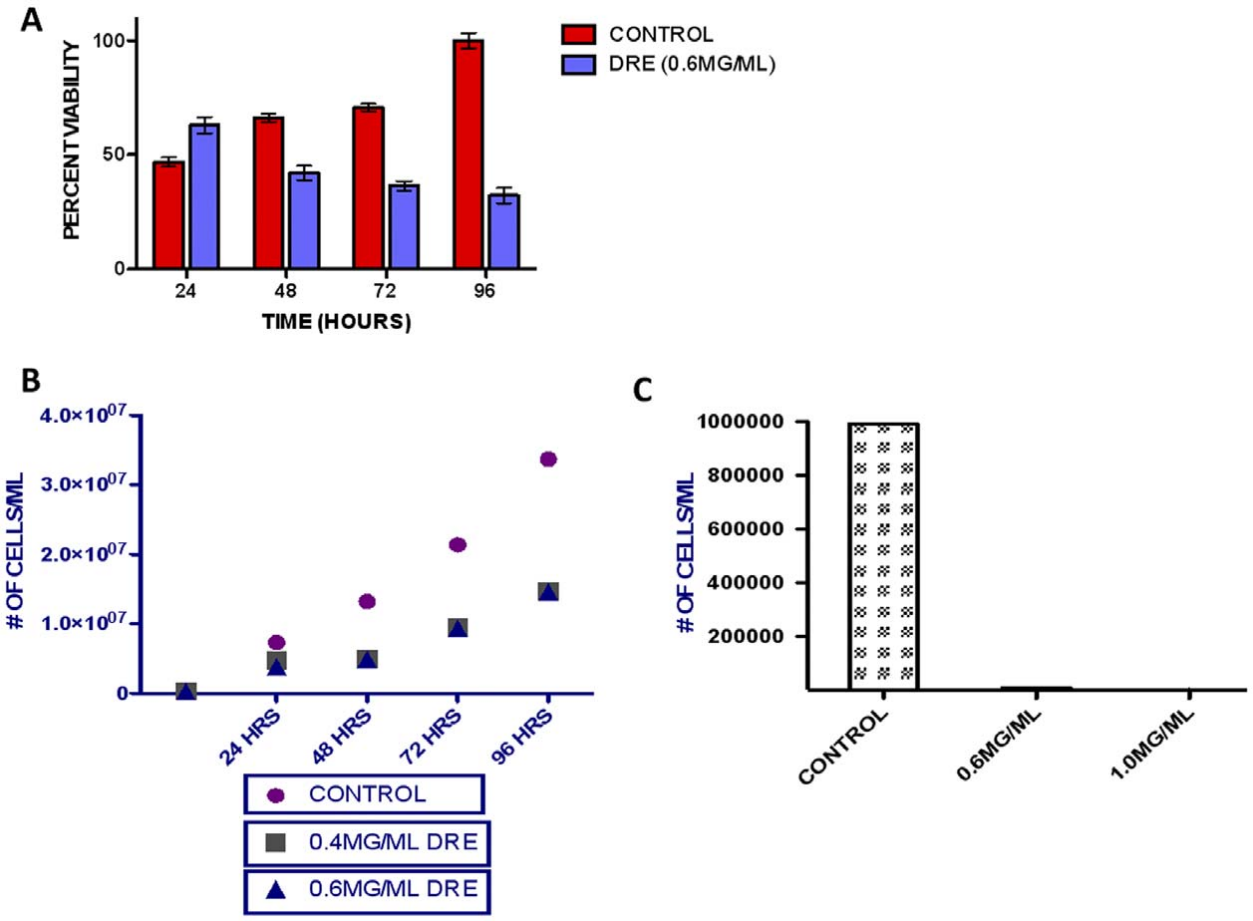
DRE reduces the viability of MV-4-11 cells in a time dependent manner. (a). MV-4-11 cells were plated in 96-well clear bottom plates and treated with DRE at indicated time points and analyzed for the ability of DRE to reduce the viability of these cells, measured by a decrease in metabolic activity. Following treatment, the WST-1 reagent was added to each well, the absorbance readings were taken at 450 nm, and expressed at a percentage of the control. The absorbance readings were analyzed using GraphPad Prism version5.0 and values are expressed as mean ± SD from quadruplicates of 3 independent experiments. (b). Following treatment with DRE for 48 hours, MV-4-11 cells were removed from media containing DRE and equal number of cells were replated in fresh media, with no extract and allowed to grow for 96 hours, observing growth every 24 hours by trypan blue exclusion assay (left). As a single dose of DRE slowed down growth of MV-4-11 cells but did not completely halt this growth, MV-4-11 cells were treated with DRE for 48 hours, then treated with a second dose of DRE after the first 48 hours. Subsequent to the second treatment, cells were observed for growth ability (right), using trypan blue exclusion assay.

To assess the effect of Dandelion Root Extract in aggressive and resistant human chronic myelomonocytic leukemia cells, MV-4-11, HL-60 and U-937 cells were treated with increasing concentrations of DRE for increasing time points. These cells were analyzed for the induction of apoptosis, using Hoechst and Annexin-V binding assay. Apoptosis was characterized by condensation of the nuclear chromatin and the externalization of the phosphatidylserine to the outer leaflet of the cell's membrane [10]. Results indicate that DRE efficiently induced apoptosis in highly aggressive and resistant leukemia cells in a dose- and time-dependent manner (Fig. 1), as an increase in the percentage of apoptotic cells corresponded to an increase in the dose and time of exposure. Another characteristic feature of apoptosis is nuclear DNA fragmentation, that can be observed after cleavage of caspase-3 [11]. To further confirm the induction of apoptosis, TUNEL assay was used to quantify DNA fragmentation in these cells after exposure to DRE. Etoposide, a known chemotherapy that targets topoisomerase II [10], was used as a positive control for DNA fragmentation. Results indicate that DRE activated the apoptotic pathways leading to late DNA fragmentation, compared to etoposide, which caused DNA fragmentation that led to the induction of apoptosis (Fig. 2). For a more quantitative assessment of the effect of DRE on CMML cells, these cells were treated with DRE and analyzed for the effect of this extract on the viability of these cells in a time-dependent manner. Results observed indicate that treatment with DRE led to a decrease in the viability of MV-4-11 cells as a function of metabolic activity, using a WST-1 cell proliferation assay (Fig. 3a). This extract led to a 60% decrease in the viability of treated cells compared to the control, untreated cells, indicating that DRE could potentially affect the metabolic activity of CMML cells, ultimately leading to cell death, observed in Fig. 1a. Since we observed an approximate decrease in viability of 60% of MV-4-11 cells 96 hours after treatment with DRE, the next step was to determine if DRE has any effect on the other 40% of cells that did not show any apoptotic morphology 96 hours after treatment. MV-4-11 cells were treated with DRE and after 48 hours the cells were removed, from media containing DRE, washed and equal numbers of cells were re-plated (for control and treated cells) in fresh media without the extract. These cells were then analyzed for their ability to revive growth after previous exposure to DRE. Although MV-4-11 cells continued to grow in the absence of DRE, it was observed that cells previously treated with DRE had reduced ability to revive growth and therefore grew at a slower pace than the cells not treated with DRE for 48 hours. More importantly, it was observed that treatment with a second dose of DRE, after 48 hours, could induce apoptosis successfully in all the cells that do not show apoptotic morphology after the first treatment (Fig. 3b).

### DRE activates the extrinsic pathway of apoptosis in CMML cells

**Figure 4:**
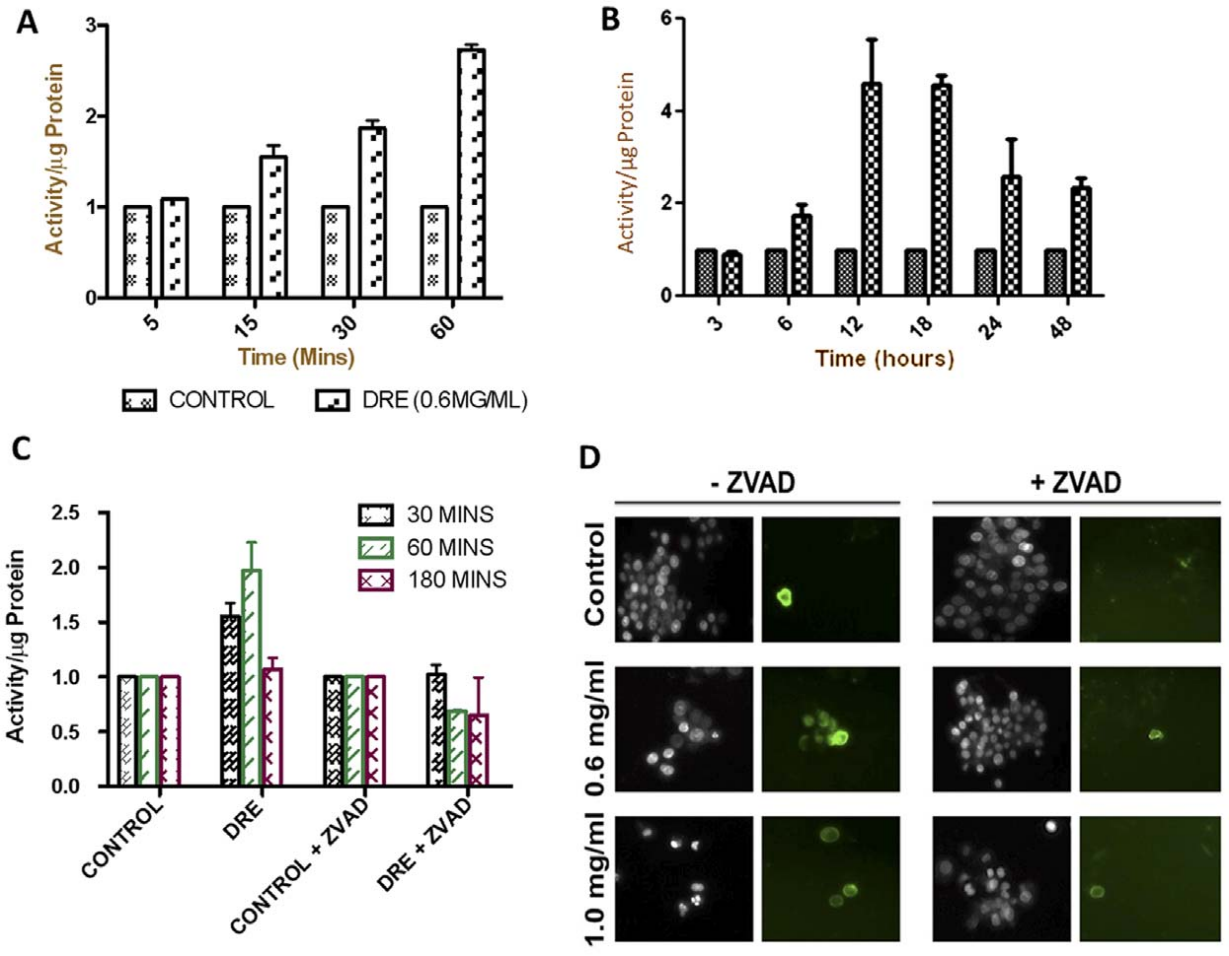
DRE activates the death-receptor-mediated extrinsic pathway of apoptosis. Following treatment with DRE, at indicated time points and concentrations, MV-4-11 cells were collected, washed and incubated with lysis buffer to obtain cell lysate. The cell lysate was incubated with caspase substrates, specific to each caspase (3,8 and 9) and incubated for an hour. Fluorescence readings were obtained using a spectrofluorometer. An average of 6 readings per well and a minimum of three wells were run per experiment. The results here are reported as activity per µg of protein (in fold) and the average of three experiments are shown (a,b). (c). Prior to DRE treatment, MV-4-11 cells were pre-treated with a pan-caspase inhibitor, Z-VAD-fmk for an hour and then treated with DRE at the indicated concentration for indicated time points. These cells were then incubated with caspase-8 substrate and fluorescence readings were obtained. These cells were also analyzed for the induction of apoptosis by Hoechst and annexin-V staining (d). Magnification: 400×.

The first step in determining the mechanism of DRE-induced apoptosis in human CMML was to assess the chronological activation of caspases in DRE-treated cells. This should give some insight to the pathway by which apoptosis is induced in these cells. In order to observe the activation of caspases, MV-4-11 cells were treated with DRE for various time points, following which, caspase activity was assayed, using substrates specific to caspases-3, -8 and -9. Results indicate that DRE rapidly activates caspase-8, followed by subsequent activation of caspase-3 at later time points (Fig. 4a and b) similar results were observed in HL-60 cells (data not shown). In order to determine if caspase activation is required for the induction of DRE-induced cell death in CMML cells, a pan-caspase inhibitor, ZVAD-fmk, was used to pre-treat the cells for an hour and then the cells were treated with DRE for different time points and assayed for caspase-8 activation. Fig. 4c confirms the inhibition of caspase-8 activation by ZVAD-fmk and this inhibition correlates with the inability of DRE to induce apoptosis in MV-4-11 cells following pre-treatment with the pan-caspase inhibitor (Fig. 4d). These results indicate that caspases are required for the apoptosis-inducing effects of DRE, more importantly, caspase-8 and -3.

**Figure 5:**
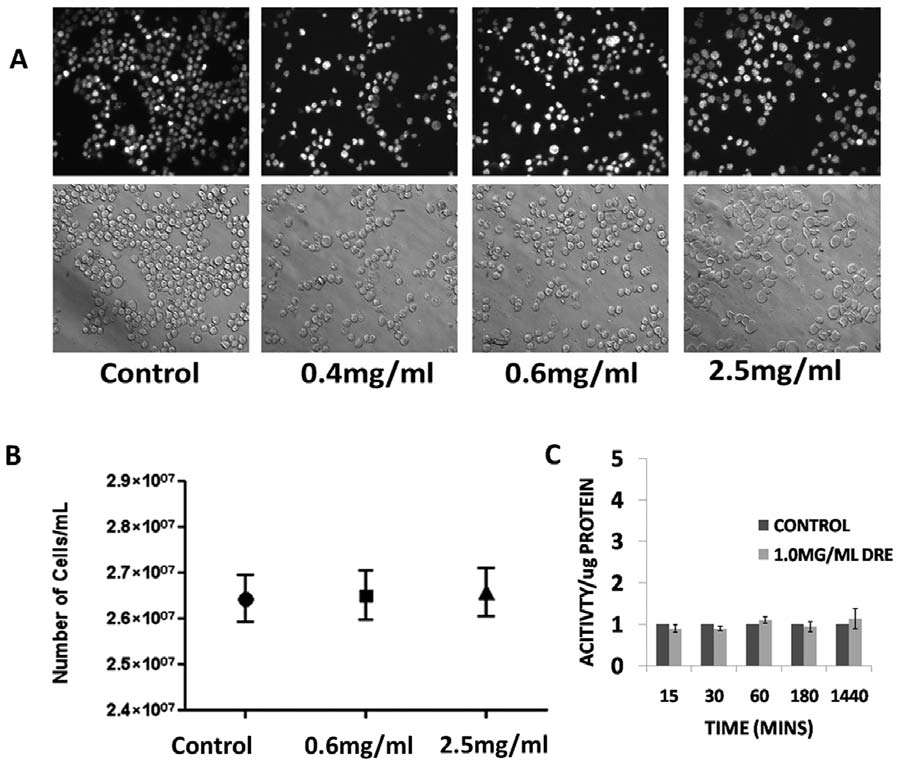
DRE required the Fas-Associated Death Domain (FADD) for its activity. (a). Dominant-negative FADD (DnFADD) cells were treated with DRE at indicated concentrations, for 96 hours and analyzed for the induction of apoptosis by nuclear condensation (Hoechst) and change in morphology (Phase contrast). Magnification: 400×. (b). Following treatment with DRE, DnFADD cells were collected and cell number was obtained using the trypan blue exclusion assay. Live cells were impermeable to the trypan blue dye. (c). DnFADD cells were treated with DRE for the indicated time points and analyzed for the activation of caspase-8, using caspase-8 specific substrate and fluorescence readings were obtained. An average of 6 readings per well and a minimum of three wells were run per experiment. The results here are reported as activity per µg of protein (in fold) and the average of three experiments are shown.

From these results, we hypothesize that DRE activates the extrinsic pathway of apoptosis. To confirm this effect on the activation of the extrinsic pathway of apoptosis, we wanted to observe the effect of DRE on cells with a dominant-negative mutation in the Fas-Associated Death Domain (FADD) protein. DnFADD cells were treated with DRE for 96 hours at increasing concentrations and analyzed for the induction of apoptosis by nuclear condensation and change in morphology. Results show that DRE did not induce apoptosis in cells with a mutant FADD domain, nor did it lead to a change in the morphology of these cells (Fig. 5a). A trypan blue exclusion assay was used to evaluate the effect of DRE on the number of cells after exposure to this extract, compared to cells not treated with DRE. In fig. 5b, we observe no significant difference between the control untreated cells and the DRE-treated cells. Furthermore, activation of caspase-8 and 3 were not observed in DRE treated DnFADD cells (Fig. 5c). These results indicate that DRE requires the Fas-Associated Death Domain for its activation of the extrinsic pathway of apoptosis.

### DRE destabilizes the mitochondrial membrane of CMML cells

**Figure 6:**
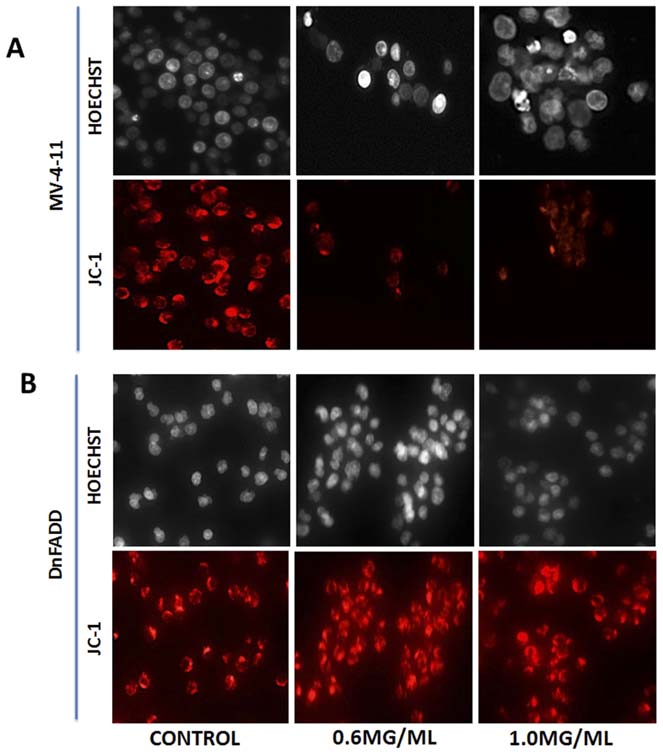
DRE destabilizes the mitochondria membrane potential of MV-4-11 cells. (a). Following treatment with DRE, MV-4-11 cells were incubated with JC-1 dye to detect the loss of mitochondrial potential (ref to Materials and Methods). Red fluorescence indicates only cells that have healthy mitochondria. The mitochondria of MV-4-11 cells are completely destabilized by DRE treatment. On the other hand, the mitochondria of DnFADD cells remained unaffected by DRE treatment (b). Magnification: 400×.

Due to the fact that the two different pathways of apoptosis can target the mitochondria, the next step was to observe the effect of DRE on the mitochondria of CMML cells affected by DRE. MV-4-11 cells were treated with DRE for 24 hours and analyzed for the destabilization of the mitochondrial membrane potential, using a cationic lipophilic dye, JC-1, which aggregates in healthy mitochondria with intact potential, to give off red fluorescence. A loss or decrease in red fluorescence is therefore indicative of a loss of mitochondrial membrane potential (MMP). Fig. 6a shows the effect of DRE on the mitochondrial membrane of MV-4-11 cells. Here, we observed a loss of red fluorescence, indicative of a loss of intact mitochondrial membrane potential, indicating that DRE has an indirect effect on the mitochondria of CMML cells, by causing the dissipation of MMP. To further implicate the extrinsic pathway of apoptosis in DRE-induced apoptosis, we wanted to observe the effect on the mitochondria of DnFADD cells treated with DRE. Fig. 6b shows the effect of DRE on the mitochondria of DnFADD cells. Here, we observed a consistency in the red fluorescence, indicating that the mitochondria of DnFADD cells remained intact and were unaffected by treatment with DRE. These results confirm that DRE does require FADD for its induction of apoptosis.

**Figure 7:**
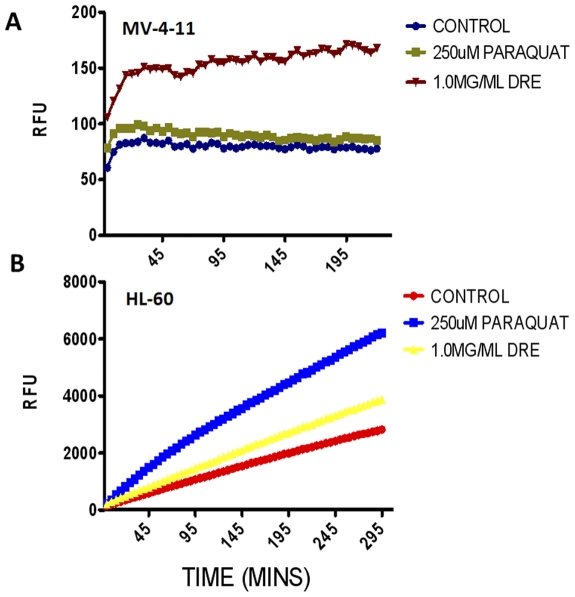
DRE increases ROS production in isolated mitochondria from CMML cells. Isolated mitochondria from (a) MV-4-11 and (b) HL-60 cells were treated directly with 1.0 mg/ml DRE and ROS production was measured using Amplex Red substrate in the presence of horseradish peroxidase (HRP). Results were compared to control untreated mitochondria and positive control, paraquat (PQ). Fluorescence readings were taken in 5 min intervals for 4 h at Ex. 560 nm and Em.590 nm and expressed as relative fluorescence units (RFU). Analysis of results were performed using GraphPad Prism version 5.0 and results shown are representative of 3 independent experiments demonstrating similar trends.

Mitochondria are considered the major players in the production of reactive oxygen species (ROS) under physiological conditions. The levels of the ROS increase drastically under pathological conditions, thereby leading to the induction of cell death [12]. Furthermore, the production of ROS is known to have dual roles; it could be the cause or effect of the induction of apoptosis. ROS has been shown to induce the activation of certain death receptors, such as Fas and TNF [13]. Therefore, these points of evidence implicate the mitochondria in the initiation and/or execution of apoptosis. Therefore, to further assess the effect of DRE on the mitochondria of CMML cells, the production of ROS was studied. To do this, the mitochondria were isolated from MV-4-11 and HL-60 cells and were treated directly with DRE. ROS production was assessed by incubation with Amplex Red dye over 4 hours, with readings taken at 5-minute intervals. Paraquat (PQ), is a known inducer of ROS production in the mitochondria [14] and this was used as a positive control of ROS production in both MV-4-11 and HL-60 cells. In both cell lines, it can be observed that there is an increase in the production of ROS in DRE-treated cells compared to the control untreated cells; in the MV-4-11 cells, this increase surpasses that of the PQ treated cells (Fig. 7a and b). Therefore, these results are indicative of the mitochondria as an indirect target of DRE.

### DRE induces pro-death autophagy in MV-4-11 cells

**Figure 8:**
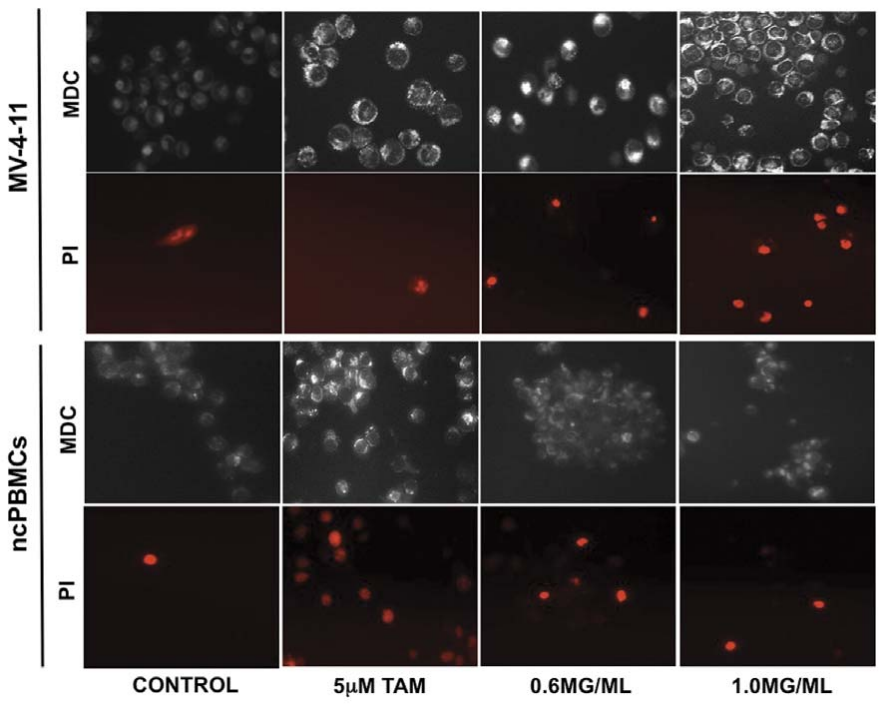
DRE triggers pro-death autophagy in MV-4-11 cells but not in non-cancerous Peripheral blood mononuclear cells (ncPBMCs). MV-4-11 cells were treated with DRE for 48 hours and analyzed for the induction of autophagy; Tamoxifen (TAM) was used as a positive control for the induction of pro-surivival autophagy. Following treatment, the cells were stained with MDC to detect autophagic vacuoles and counterstained with PI to detect cell death. Fluorescence images were captured. Magnification: 400×.

Autophagy, also known as “self-eating”, is an evolutionary conserved process that is known to play a significant role in the maintenance of cellular homeostasis. It is the primary degradation process by which cells can get rid of long-lived or defective proteins, as well as, defective organelles [15]. It is reported to play a dual role, as it supports cell survival and cell death (in extreme cases of “self-eating”) [16]. Various cellular stressors, such as hypoxia, starvation, protein aggregation and ROS production, usually induce this process. The presence of these stressors leads to the formation of the autophagosome, a double membrane vesicle, around the protein or organelle to be degraded. The autophagosome will fuse to the lysosome and its contents can be degraded [17], [18]. In order to assess the induction of autophagy in CMML cells, MV-4-11 cells were treated with DRE for 48 hours, then stained with monodansylcadaverine (MDC) dye and counterstained with propidium iodide to observe cell death. Tamoxifen (TAM), a known inducer of pro-survival autophagy [19], was used as a positive control. Results indicate that, unlike TAM, DRE does induce a pro-death form of autophagy, as characterized by the bright punctate MDC staining, as well as the propidium iodide positive staining, in DRE-treated cells, comparable to the TAM-treated cells. These cells were more brightly stained than the control untreated cells (Fig. 8; top panel).

### DRE is selective to CMML cells, with little toxicity on normal, non-cancerous cells

**Figure 9:**
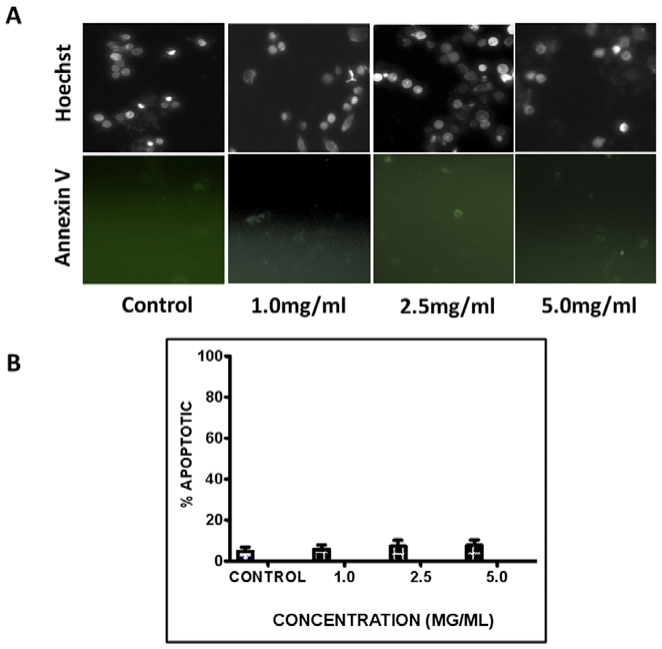
DRE does not target normal non-cancerous peripheral blood mononuclear cells (ncPBMCs). (**a**). **nc**PBMCs were isolated from healthy volunteers and plated in six-well plates. These cells were treated with DRE at increasing concentrations for 96 hours and analyzed for the induction of apoptosis by nuclear condensation (Hoechst) and externalization of the phosphatidylserine (Annexin-V binding). Magnification: 400×. (b). Results from three different experiments were quantified to determine the percentage of apoptosis occurring in ncPBMCs treated with DRE.

Dandelions have been used for centuries for nourishment and therapy, with few reports of little to no toxicity, due to the lack of toxins and alkaloids present in this plant. High doses have been reported to cause allergic contact dermatitis, although this was shown in studies using dandelion extract as a topical treatment [6]. In order to assess toxicity of DRE in the lab, non-cancerous peripheral blood mononuclear cells (ncPBMCs) were isolated from the blood of apparently healthy volunteers, according to a previously published protocol [20]. These cells were plated into six-well culture plates and treated with DRE at increasing concentrations for 96 hours, then analyzed for the induction of apoptosis. Results show that DRE does not induce apoptosis in ncPBMCs, even at high doses (Fig. 9a and b). As ncPBMCs are not actively proliferating in culture, some isolated PBMCs were pre-incubated with concanavalin A (conA), a plant mitogen known to stimulate T cell proliferation *in vitro* [21]. These cells were then exposed to DRE treatment and analyzed for the induction of apoptosis. As with the ncPBMCs not pre-treated with conA, those pre-incubated with conA and subsequently treated with DRE remained unsusceptible to DRE-induced apoptosis (data not shown). These results have also been shown in other non-cancerous cell lines, for example, normal human fibroblasts (NHFs) [8]. To further evaluate the selective induction of cell death in ncPBMCs, these cells were treated with DRE for 48 hours and stained with MDC and PI to assess the induction of pro-death autophagy, using tamoxifen as a positive inducer of autophagy. Figure 8 (bottom panel) indicates that tamoxifen effectively induced autophagy in ncPBMCs, ultimately leading to the cell death, observed with propidium iodide staining. Importantly, there was no induction of autophagy in these cells, as compared to the control, untreated cells. These DRE treated cells were also impermeable to propidium iodide, confirming the lack of cell death in ncPBMCs treated with DRE. These results suggest that DRE is non-toxic to non-cancerous blood cells, making it selective to CMML cells.

## Discussion

The poor prognosis for Chronic Myelomonocytic Leukemia (CMML) patients is a major indication that there is a serious need for a more effective and non-toxic alternative to the conventionally available forms of chemotherapy and surgical procedures [4]. With the introduction of natural products, not only as sources of nourishment, but also for their therapeutic benefits, it is therefore necessary to study the vast array of natural products as non-toxic and less expensive alternatives for the treatment of CMML. Apoptosis and autophagy, two necessary modes of programmed cell death, are important mechanisms, which cells utilize for the maintenance of cellular homeostasis [22]. Cancer cells, however, have developed mechanisms to evade these programs, so as to enable enhanced proliferation, aggressiveness and resistance [23], [24]. In this study, we demonstrate the selective efficacy of dandelion root extract in inducing apoptosis and autophagy in highly aggressive and resistant CMML cell lines.

We observed the induction of apoptosis in three of the CMML cell lines used for this study (Fig. 1). More importantly, this effect was selective, as non-cancerous PBMCs and NHFs remained unsusceptible to DRE-induced apoptosis (Fig. 9a and b).

Although various extracts of dandelions have been used for centuries for the treatment of various diseases, there have been very few scientific studies done to ascertain the mechanism by which these extracts act and although some of the compounds present in dandelion extracts have been isolated and identified, they still are not fully characterized [6], [7]. The major aim of the study was therefore to determine the mechanism of DRE-induced cell death in CMML cells.

In this study, we report a rapid activation of caspase-8 (within minutes) and subsequent activation of caspase-3 in human CMML cells, indicative of a speedy activation of the death receptor mediated extrinsic pathway of apoptosis (Fig. 4a and b). This activation has been shown to be necessary for the induction of apoptosis observed after DRE treatment, as the inhibition of caspase activity, using a pan-caspase inhibitor, ZVAD, prevented the induced cell death observed (Fig. 4c and d). These results suggest that caspases, more importantly, caspase-8 and 3, are required for the induction of apoptosis by DRE. The Fas-Associated Death Domain (FADD), as well as other death domains, are highly important for activation of caspase-8 through extrinsic apoptosis [25]. The binding of the death ligand to its corresponding death receptor leads to the recruitment of specific receptor death domains for the formation of the death inducing signalling complex (DISC) [26]. The absence of FADD should therefore prevent the activation of the extrinsic pathway of apoptosis through inhibition of the activation of caspase-8 [27]. To further confirm the effect of DRE on the extrinsic pathway of apoptosis, DnFADD cells were treated with DRE and analyzed for apoptosis induction. According to our results, no nuclear condensation and morphological changes were observed with the treatment of DRE. There was no change in the number of cells treated with DRE, compared to the control untreated cells, suggesting that DRE did not have a significant effect on the cells with a truncated FADD protein (Fig. 5a and b). Furthermore, these cells were not susceptible to DRE-induced rapid activation of caspases, specifically caspase-8 (Fig. 5c). As we observed late activation of caspase-3, we wanted to observe the connection between the intrinsic and the extrinsic pathway of apoptosis. It is known that the activation of caspase-8 could yield two main results. First, the activated caspase-8 could lead directly to the activation of the effector caspase, caspase-3 or it could lead to mitochondrial changes, through the cleavage of pro-apoptotic protein, Bid. The truncation of Bid causes mitochondrial membrane destabilization, which leads to the release of pro-apoptotic proteins for the activation of caspase-3 [26]. In this study, we observed the destabilization of the mitochondrial membrane potential 24 hours after treatment with DRE. This destabilization of mitochondrial membrane potential was only observed in MV-4-11 cells, not in DnFADD cells (Fig. 6a and b). These results suggest that DRE indirectly targets the mitochondria after the activation of the extrinsic pathway of apoptosis. It confirms the requirement of the adapter domain for DRE-induced apoptosis, as we do not observe a similar loss of mitochondrial membrane potential in DnFADD cells treated with DRE. As the mitochondria are major players in the production of ROS [28], the next step was to observe the effect of DRE on the production of ROS. Results confirm the production of ROS from isolated mitochondria (Fig. 7a and b), further confirming the effect of DRE on the mitochondria of CMML cells.

The integrity of the mitochondria is essential for the maintenance of cellular homeostasis and defects in the mitochondria caused by treatment with DRE could provide sufficient signals for the induction of programmed cell death type II, also known as Autophagy, a process induced by the presence of defective organelles, proteins and starvation [29]. Autophagy has been shown to play a significant role in cancer survival and its role in programmed cell death has been controversial. In response to stressors, cells undergo “self-digestion” as a means of temporary survival, where macromolecules are digested in order to provide an alternate energy source. However, excessive exposure to stressors could lead to excessive autophagy, ultimately resulting in cell death [18], [30]. The loss of mitochondrial membrane potential, observed after treatment with DRE, therefore led us to determine if the stress induced by our extract, leading to mitochondrial membrane destabilization was a sufficient signal for the induction of autophagy. Our results indicate that treatment with DRE triggered the induction of a pro-death form of autophagy in MV-4-11 cells (Fig. 8; top panel). Comparing this to treatment with tamoxifen, which is known to induce pro-survival in cancer cells, treatment with DRE not only triggered autophagy, comparable to TAM, the cells were also propidium iodide positive, indicative of cell death. Moreover, ncPBMCs stained with MDC and PI showed no induction of autophagy in these cells after DRE treatment (Fig. 8; bottom panel). These results suggest that DRE is effective in selectively inducing both forms of programmed cell death in human CMML cells.

In conclusion, Dandelion Root Extract has shown selective efficacy in inducing two forms of programmed cell death in highly aggressive and resistant CMML cell lines. The rapid activation of caspase-8 not only activated the extrinsic pathway of apoptosis, but also triggered pro-death autophagy selectively in these cells, suggesting that this extract has components that enhance its selective efficacy in targeting CMML cells. These results indicate that within the vast array of available natural products and compounds, there are non-toxic alternatives to conventional chemotherapy that are safe and effective.

## Materials and Methods

### Standardized Dandelion Root Extraction

Freshly obtained Dandelion roots (from open grassy areas) were processed according to a previously published protocol [9]. The roots were thoroughly washed with distilled water several times. One hundred grams of Dandelion roots were homogenized in 200 ml of distilled water at room temperature using a domestic blender. Total homogenate was filtered through a NITEX nylon mesh filter (LAB PAK; Sefar BDH Inc. Chicoutimi, Quebec CA) and the filtrate was spun down, 8000× *g* for 5 min at 25°C. The supernatant was filtered using 0.45 µm filters, followed by lyophilisation. The dry powder (% yield: 7.34%) was reconstituted in water to give a stock solution of 100 mg/ml DRE. This was then filtered through 0.22 µm filters and used in the treatment of Chronic Myelomonocytic leukemia cells.

### Cell Culture and Treatment

Human CMML cell lines (MV-4-11, HL-60 and U-937 cells), as well as a dominant negative FADD Jurkat cell line was purchased from ATCC (Manassas, VA.). MV-4-11 and HL-60 cells were cultured in Iscove's Modified Dulbecco's Medium, supplemented with 15% fetal bovine serum (FBS) and 40 µg/ml gentamicin (Life Technologies, Mississauga, Ontario). The DnFADD cells were cultured in RPMI-1640 medium supplemented with 15% FBS and 40 µg/ml gentamicin. These cells were grown and maintained in an incubator set at 37°C with an atmosphere containing 5% CO_2_ and 95% humidity. Human nucleated blood cells were purified from whole blood obtained from healthy volunteers, modified from a previously published protocol [31] and as approved by the University of Windsor ethical committee, REB# 04-060. Whole blood (12 ml) was collected into a BD Vacutainer CPT Tube (Cell Preparation Tube) obtained from Becton Dickinson (Franklin Lakes, N.J.). The whole blood was spun down in a tabletop low-speed centrifuge at 2900× *g* for 30 min at 25°C. The red blood cells went through the polyester gel and the top layer containing mononuclear cells, platelets and plasma was collected. These cells were kept in the same incubator as the CMML cells (37°C, 5% CO_2_ and 95% humidity) and cultured in AIM-V medium from Invitrogen (Burlington, ON). For the induction of apoptosis and autophagy by treatment with Dandelion Root Extract (DRE), Cells were grown to 50–70% confluence and then treated either with different concentrations of lyophilized extract (0.6 mg/ml to 5.0 mg/ml). At different times after treatment, cells were analyzed for apoptotic markers as described below.

### Cell staining

#### Trypan Blue Exclusion Assay

To examine the ability of MV-4-11 cells to revive growth after previous exposure to DRE, these cells were treated with DRE for 48 hours, after which the cells were removed from media containing extract and equal number of cells were replated and examined every day. After treatment, a cell suspension was added (1∶1) to Trypan Blue stain (Life Technologies). Using a hemacytometer (Fisher Scientific, Horsham, Pa.), live cells (trypan blue negative) were counted three times, calculated and tabulated as number of cells per ml using GraphPad Prism 5.0 288 software.

#### Hoechst Staining

Cells were grown and treated, and then stained with cell-permeable Hoechst 33342 (Molecular Probes, Eugene, Ore.), at a final concentration of 10 µM and incubated for 10 min at 37°C. The cells were then examined under a fluorescent microscope (Leica DM IRB, Germany) and fluorescent pictures were taken. Five fields of fluorescent pictures were used to count apoptotic versus live cells (where brightly stained cells with condensed nuclei were considered apoptotic). These results were then calculated and tabulated as percentage of apoptotic cells using GraphPad Prism 5.0 288 software.

#### Annexin-V Binding Assay

To confirm the induction of apoptosis by staining for phosphotidylserine exposed on the outer leaflet of the plasma membrane (a characteristic feature of apoptosis), CMML cells were grown and treated with different concentrations of DRE. The cells were collected after given periods as indicated, washed in phosphate-buffered saline (PBS) and resuspended in annexin-V binding buffer (10 mM HEPES/NaOH pH 7.5, 140 mM NaCl, 2.5 mM CaCl2), containing 1∶50 annexin-V Alexa Fluor 488 conjugate (catalog no. A13201, Molecular Probes) for 15 min at room temperature. Cells were then examined under a fluorescent microscope (Leica DM IRB), and pictures were taken. All pictures were processed using Adobe Photoshop 7.0 software.

#### Mitochondrial Membrane potential

JC-1 Mitochondrial Membrane Potential Detection Kit was applied to monitor mitochondrial membrane destabilization in cells undergoing apoptosis. In non-apoptotic cells with healthy mitochondria, JC-1 (5, 5′, 6, 6′-tetrachloro-1, 1′, 3, 3′ tetraethylbenzimidazolylcarbocyanine iodide) (Catalog. No. M34152, Burlington, ON) exists as a monomer in the cytosol and also accumulates as aggregates in the mitochondria which fluoresce red. In apoptotic and necrotic cells, JC-1 exists in monomeric form, diffused in the cytosol where it fluoresces green. MV-4-11 and DnFADD cells were collected after treatment and incubated with 100 µL per ml JC-1 dye for 15 min at 37°C. The cells were examined under fluorescent microscope (Leica DM IRB) and fluorescent pictures were taken. All pictures were processed using Adobe Photoshop 7.0 software.

#### Monodansylcadaverine (MDC) Staining

Monodansylcadaverine (MDC) (Sigma-Aldrich Canada, Mississauga, ON, Canada) was used to visualize autophagic vacuoles. CMML cells were plated in six-well plates and treated with different concentrations of DRE for 48 hours. After treatment, cells were incubated with 0.1 mM MDC for 30 minutes. Fluorescent pictures were acquired at 400× magnification on a Leica DM IRB inverted fluorescence microscope.

#### Propidium Iodide Staining

Cell death was detected by cell lysis observed by staining with propidium iodide (PI) dye (Sigma Aldrich, Canada, Missisauga, ON. Canada). After incubating with MDC dye for 15 minutes, cells also co-stained with PI at a concentration of 1.0 µg/mL for the last 15 minutes of incubation with MDC. Following incubation, the cells were visualized and images were taken using a fluorescence microscope at 400× objective.

#### TUNEL staining

After treating MV-4-11 cells with DRE for the indicated time points, the TUNEL assay was performed according to the manufacturer's protocol (Molecular Probes, Eugene, OR) and a previously published protocol [32], in order to detect DNA damage. Cells were treated with DRE or VP-16 (as a positive control) at indicated concentrations and time points and analyzed for the fragmentation of DNA. Following treatment, cells were fixed by suspending them in 70% (v/v) ethanol and stored at −20°C overnight. The sample was then incubated with a DNA labeling solution (10 µL reaction buffer, 0.75 µL TdT enzyme, 8 µL BrdUTP, 31.25 µL of dH2O) for 1 hour at 25°C. Each sample was exposed to an antibody solution (5 µL Alexa Fluor 488 labeled anti-BrdU antibody and 95 µL rinse solution). The cells were incubated with the antibody solution for 20 minutes and pictures were taken using a fluorescent microscope (Leica DM IRB, Germany).

### Cell viability Assay

To examine the viability of MV-4-11 cells after treatment, cells were incubated with cell proliferation reagent WST-1 (Catalog No. 05 015 944 001, Roche Diagnostics) for 4 h at 37°C, following treatment with DRE at indicated doses and time points, using manufacturers protocol. WST-1 works by reacting with the mitochondrial succinate-tetrazolium reductase forming the formazan dye. The WST-1 reagent produces a water-soluble formazan product [33]. Fluorescence readings were obtained at 450 nm using a Perkin Elmer Victor Fluorescence machine. Viability readings were analyzed using GraphPad Prism 5.0 288 software and expressed as percentages of the control untreated groups.

### Caspase Activity

The Caspase assays were performed using a previously published method [34]. To determine caspase activity, the total protein from MV-4-11, HL-60 and DnFADD cell lysates were incubated with the fluorogenic substrates corresponding to the substrate cleavage site, specific for each caspase, DEVD-AFC for Caspase-3 and IETD-AFC for Caspase-8. The fluorescence was measured at an excitation wavelength of 400 nm and emission wavelength of 505 nm using a Spectra Max Gemini XS (Molecular Devices, Sunnyvale, California). Caspase activity was calculated as activity per µg protein and protein concentration was determined with BioRad protein assay reagent (BioRad, Mississauga, Ontario) using bovine serum albumin (BSA) as a standard. Readings were analyzed using GraphPad Prism 5.0 288 software. To further confirm the role of caspases in apoptosis induction by DRE, the zVAD-fmk caspase inhibitor (Catalog No. 219007, EMD4Biosciences, San Diego, California), was incubated with MV-4-11 cells an hour before treatment described above. Some cells were collected, washed in PBS and stained with Hoechst 33342. Others were analyzed for the activation of caspases, using fluorogenic substrates described above, after DRE treatment.

### Mitochondrial Isolation and measurement of ROS production

Mitochondria were isolated from untreated MV-4-11 and HL-60 cells for analysis of ROS production. Cells were washed twice in cold PBS, resuspended in hypotonic buffer (1 mM EDTA, 5 mM Tris–HCl, 210 mM mannitol, 70 mM sucrose, 10 µM Leu-pep, 10 µM Pep-A, and 100 µM PMSF) and immediately homogenized, and centrifuged at 600× g for 5 minutes at 4°C. The supernatant was centrifuged at 15,000× g for 15 minutes at 4°C. The resulting cytosolic supernatant was discarded and the mitochondrial pellet was resuspended in cold hypotonic buffer.

To measure the production of ROS, Amplex Red (Molecular Probes, Eugene, OR) was used. The isolated mitochondria were resuspended in cold hypotonic buffer and 20 µg of protein was added to wells of a 96-well opaque plate. Treatment was done with 1.0 mg/ml DRE and as a positive control, 250 µM paraquat (PQ) (Sigma Aldrich, Missisauga, ON. Canada) was used for treatment. Amplex Red reagent was added to each well for a final concentration of 50 µM; horseradish peroxidase (HRP) was added in the ratio of 6units per 200 µL. Fluorescence readings were taken every 5 minutes for a total time of 4 hours at Ex. 560 nm and Em. 590 nm on a spectrofluorometer (SpectraMax Gemini XS, Molecular Devices, Sunnyvale, CA). The readings were analyzed using GraphPad Prism 5.0 288 software and expressed as relative fluorescence units (RFU).
